# The impact of child mortality on fertility in South Africa: Do child support grants and antiretroviral treatment matter?

**DOI:** 10.1371/journal.pone.0284032

**Published:** 2023-04-04

**Authors:** Mashudu Lucas Bidzha, Leigh F. Johnson, Rob E. Dorrington, Nicholas Ngepah, Talita Greyling

**Affiliations:** 1 School of Economics, University of Johannesburg, Auckland Park, South Africa; 2 Centre for Infectious Disease Epidemiology and Research, University of Cape Town, Cape Town, South Africa; 3 Centre for Actuarial Research, University of Cape Town, Cape Town, South Africa; Sri Lanka Institute of Information Technology, SRI LANKA

## Abstract

This paper investigates the effect of under-five mortality, child support grant (CSG) coverage and the rollout of antiretroviral therapy (ART) on fertility in South Africa. The study employs the quality-quantity trade-off framework to analyse the direct and indirect factors affecting fertility using the two stage least squares fixed effects instrumental variable approach. The analysis uses balanced panel data covering nine provinces from 2001–2016. This period was characterised by significant increases in the child support grant coverage and ART coverage. Furthermore, this period was characterised by a significant decline in the under-five mortality rate. We find no evidence to support the hypothesis that increases in the CSG coverage are associated with an increase in fertility. This finding aligns with previous literature suggesting that there are no perverse incentives for childbearing associated with the child support grant. On the other hand, results indicate that an increase in ART coverage is associated with an increase in fertility. Results also show that a decrease in under-five mortality is associated with a decline in fertility over the sample period. HIV prevalence, education, real GDP per capita, marriage prevalence and contraceptive prevalence are also important determinants of fertility in South Africa. Although the scale up of ART has improved health outcomes, it also appears to have increased fertility in HIV-positive women. The ART programme should therefore be linked with further family planning initiatives to minimise unintended pregnancies.

## 1. Introduction

The Sustainable Development Goals call for major reductions in under-five mortality, poverty, gender inequality, HIV/AIDS and unmet need for family planning–all of which are thought to be important determinants of fertility. Few studies have investigated the effect of progress towards these goals on fertility in the South African context. Over the period 2001 to 2016, there has been a decline in the under-five mortality rate, an increase in child support grant (CSG) coverage and increase in antiretroviral treatment coverage in South Africa. These changes occurred while low levels of fertility prevailed in South Africa compared to the rest of the African continent. A better understanding of the factors behind the prevailing fertility levels would be useful not only for South Africa, but also more generally. South Africa is a unique context in which to analyse the determinants of fertility given these prevailing factors that have received limited scrutiny in the economic literature.

The effect of under-five mortality reductions on fertility is one of the key mechanisms emphasised in the economic growth literature explaining demographic transitions [1, 2]. Lorentzen et al. [3] suggest that health (mortality) affects economic growth through various channels and one of them is its effects on fertility. Theoretically, increased fertility acts to reduce the influence of health capital on economic growth [4].

Several studies have assessed the impact of under-five mortality on fertility and the results are mixed. Some studies find that a decrease in under-five mortality is associated with a decline in fertility [1, 3, 5–9]. On the other hand, some studies find no statistically significant relationship between under-five mortality and fertility [10–12].

In this paper, we argue that the mixed results may be an indication that the relationship between under-five mortality and fertility is context-specific, and as such requires country-specific analysis. We also argue that the mixed results suggest the presence of omitted variables bias. This is in line with observations by Aksan and Chakraborty [5] and Burger et al. [10] who pointed out that the effect of a decline in under-five mortality on fertility also depends on morbidity effects in both adults and children, and whether the prevalence or case fatality rate declines. For example, Okada [13] found that women living with HIV tend to have fewer children. Furthermore, Young [14] found that the HIV epidemic has lowered fertility in sub-Saharan Africa. This paper builds on these insights and controls for the effects of HIV/AIDS treatment and CSG coverage.

Studies about the determinants of fertility in South Africa based on econometric analysis are scarce [10, 15]. This paper adds new within-country evidence to the few existing studies that have so far investigated the determinants of fertility in South Africa using regression analysis.

South Africa has a well-functioning CSG system. The provision of the CSG in South Africa continues to incite debates in the public discourse about the perverse incentives for childbearing associated with the grant [15–19]. Specifically, it is often suggested that the CSG encourages women in poor households to have more children in order to increase their income. Although there are few studies in South Africa that have evaluated the effect of the CSG on teenage pregnancy [15–18], only one study [19], to the best of our knowledge, has assessed the effect of CSG on fertility over all ages. However, this study relied on propensity score matching, a technique that can introduce bias and model dependence [20].

There is also a lack of attention given to the effect of antiretroviral treatment (ART) on fertility in the economic literature when evaluating determinants of fertility. Despite epidemiological data showing the effect of ART on fertility at the individual level, no study, to the best of our knowledge, has examined the effects of ART coverage on population-level fertility in the economic literature. Several epidemiological studies support the hypothesis that an increase in the ART coverage is associated with an increase in fertility [21–24]. According to Makumbi et al. [22], the advent of ART has improved the general health of people living with HIV/AIDS and may increase sexual activity leading to an increase in fertility.

The study employs the two stage least squares fixed effects instrumental variable (2SLS-FE-IV) approach to analyse balanced panel data covering nine provinces from 2001–2016. The 2SLS-FE-IV estimator is robust to various estimation problems that often affect empirical analysis, including endogeneity from omitted variables and reverse causality [25]. As observed by Hansen and Lønstrup [12], the use of data from a single country makes the study less susceptible to unobserved time varying effects such as culture changes and data quality, across observed units, compared to cross-country analyses. To the best of our knowledge, this is the first study to explore the effects of CSG and ART coverage on fertility in a multivariate panel data setting in South Africa. This has been made possible by the richness of the provincial level data estimates from the Thembisa model version 4.3 [26] and the CSG data from the South African Social Security Agency (SASSA) [27].

This paper investigates the role of under-five mortality, child support grant uptake and ART rollout as drivers for change in fertility. In doing so, the paper tests the hypothesis that a decrease in under-five mortality leads to a decline in fertility. Furthermore, the paper tests the hypotheses that CSG coverage leads to increases in the total fertility rate and that ART rollout leads to increases in the total fertility rate. The remainder of the paper is organised as follows. First, we provide an overview on under-five mortality, total fertility rate, ART coverage and CSG coverage in section 2. This is followed by the conceptual framework on the determinants of fertility in section 3. A review of the empirical literature is provided in section 4 and this is followed by a brief exposition of the empirical model and the estimation approach in section 5. Section 6 focuses on data selection and sources of data. Thereafter, a discussion of the empirical results is provided in section 7 and finally some discussion and concluding remarks in section 8.

## 2. Under-five mortality, fertility, antiretroviral coverage and child support grant coverage in South Africa: An overview

**Fig 1:**
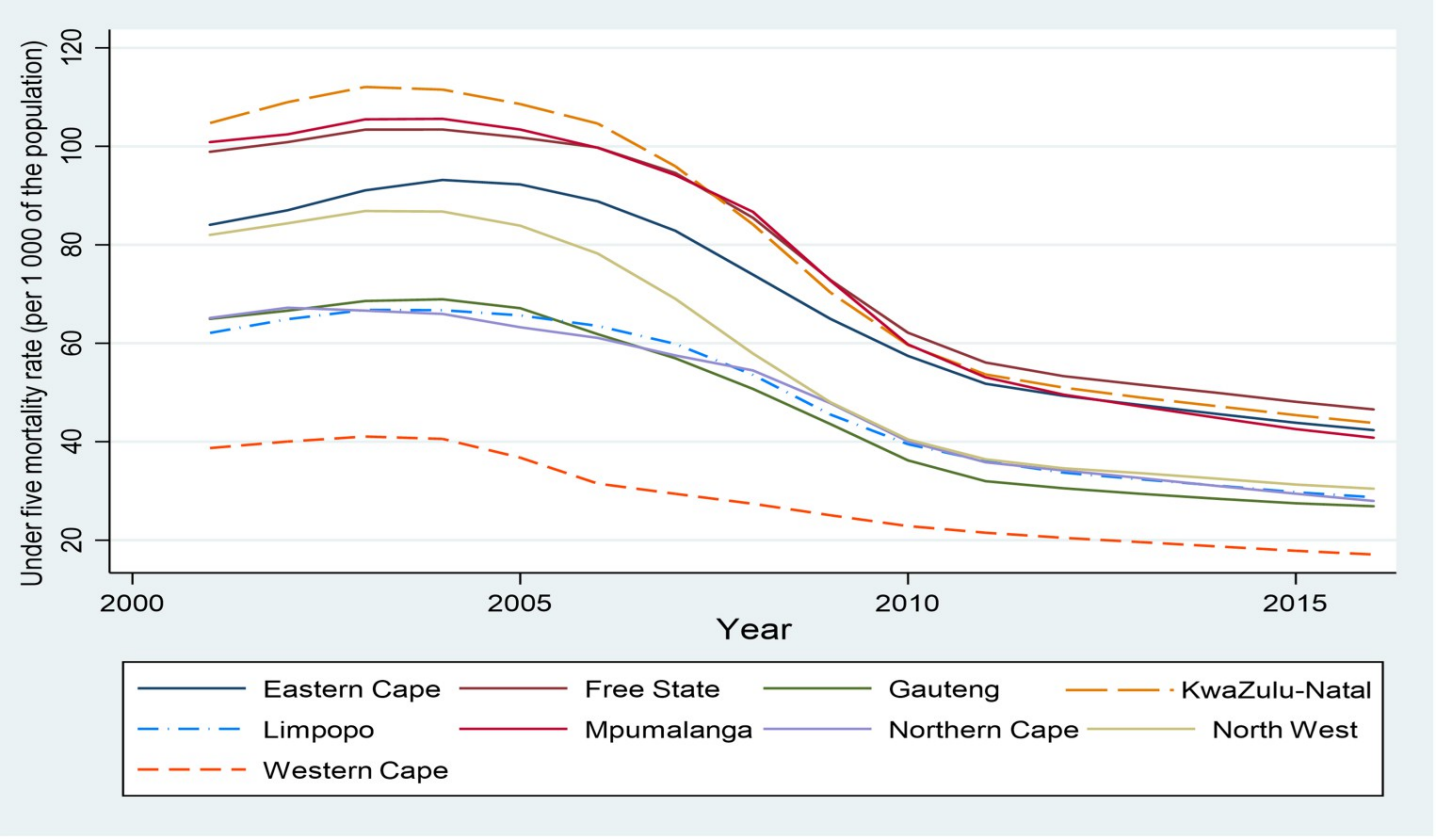
Provincial under-five mortality rate over time, 2001–2016. Source: Authors’ calculation using data from the Thembisa model by Johnson and Dorrington *[26]*.

**Fig 2:**
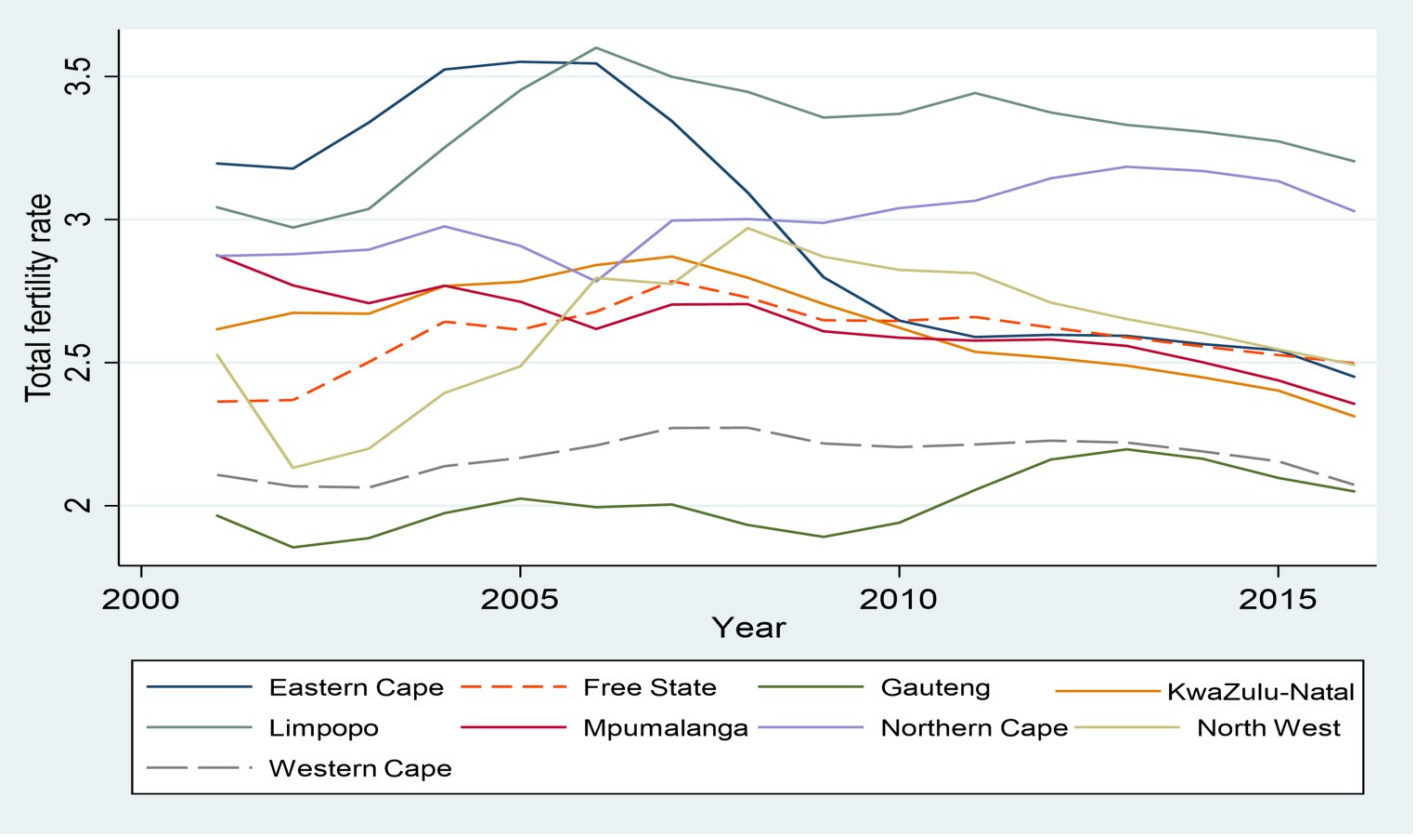
Provincial TFR over time, 2001–2016. Source: Authors’ calculation using data from the Thembisa model by Johnson and Dorrington [26].

Fig 1 shows the declining trend in provincial under-five mortality rate over the period 2001 to 2016 while Fig 2 shows the trend in total fertility rate (TFR) in South Africa by province over the same period. Under-five mortality declined in unison for all provinces (Fig 1), while the trends in the total fertility rate were different for most provinces (Fig 2). Over the period 2001 to 2016, under-five mortality rates peaked between 2003 and 2004, followed by a rapid decline until 2011 and thereafter declined more gradually. In 2016, the Free State province had the highest under-five mortality rate of 46.6 per thousand live births while the Western Cape had the lowest under-five mortality rate of 17.1 per thousand live births. A recent study by Johnson et al. [28] found that declines in under-five mortality were largely driven by the prevention of mother-to-child transmission (PMTCT) programmes and to some extent by the paediatric ART programme. In addition, part of the decline could be due to increased vaccination coverage [29, 30].

Since 2001, South Africa has experienced a small decrease in fertility with the total fertility rate declining from 2.50 in 2001 to 2.35 in 2016 [26]. TFR at a point in time is the average number of children women would have by the time they reach 49 years if the women experienced the age-specific fertility rates measured at that point in time [26]. In terms of the provinces, Limpopo had the highest fertility rate of 3.20 in 2016 while Gauteng had the lowest fertility rate of 2.05.

**Fig 3:**
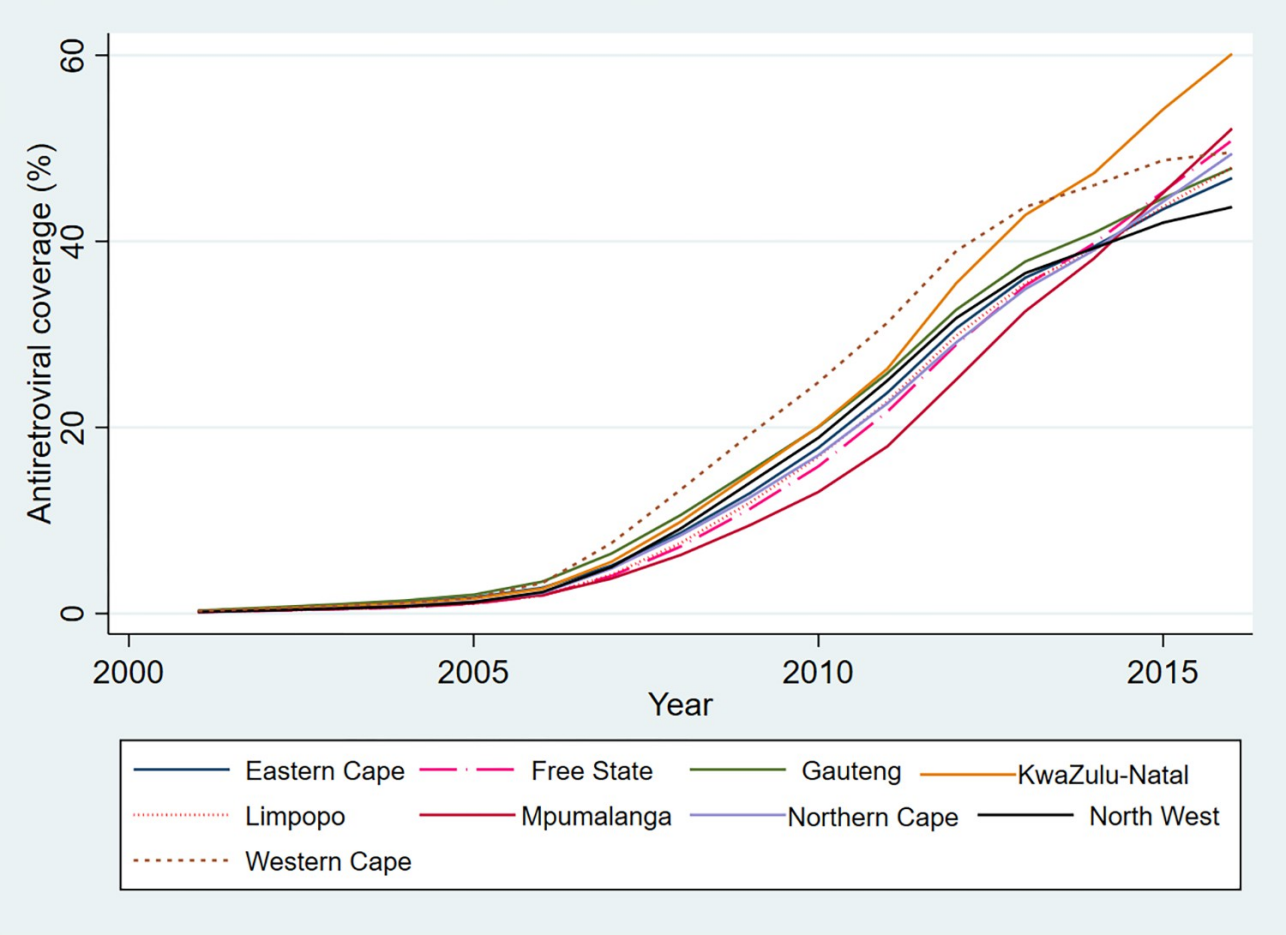
Provincial ART coverage over time, 2001–2016. Source: Authors’ calculation using data from the Thembisa model by Johnson and Dorrington [26].

At the end of the 2016/17 financial year, there were 3.8 million people receiving ART in the public sector in South Africa [31]. Globally, South Africa has the largest ART programme, accounting for 20% of people on ART [32]. South Africa’s ART coverage grew from 0.2% in 2001 to 53.5% in 2016 [26]. ART coverage is defined as the number of individuals receiving antiretroviral treatment at a point in time divided by the number of individuals needing treatment [26]. Fig 3 shows ART coverage in South Africa by province over the period 2001 to 2016.

**Fig 4:**
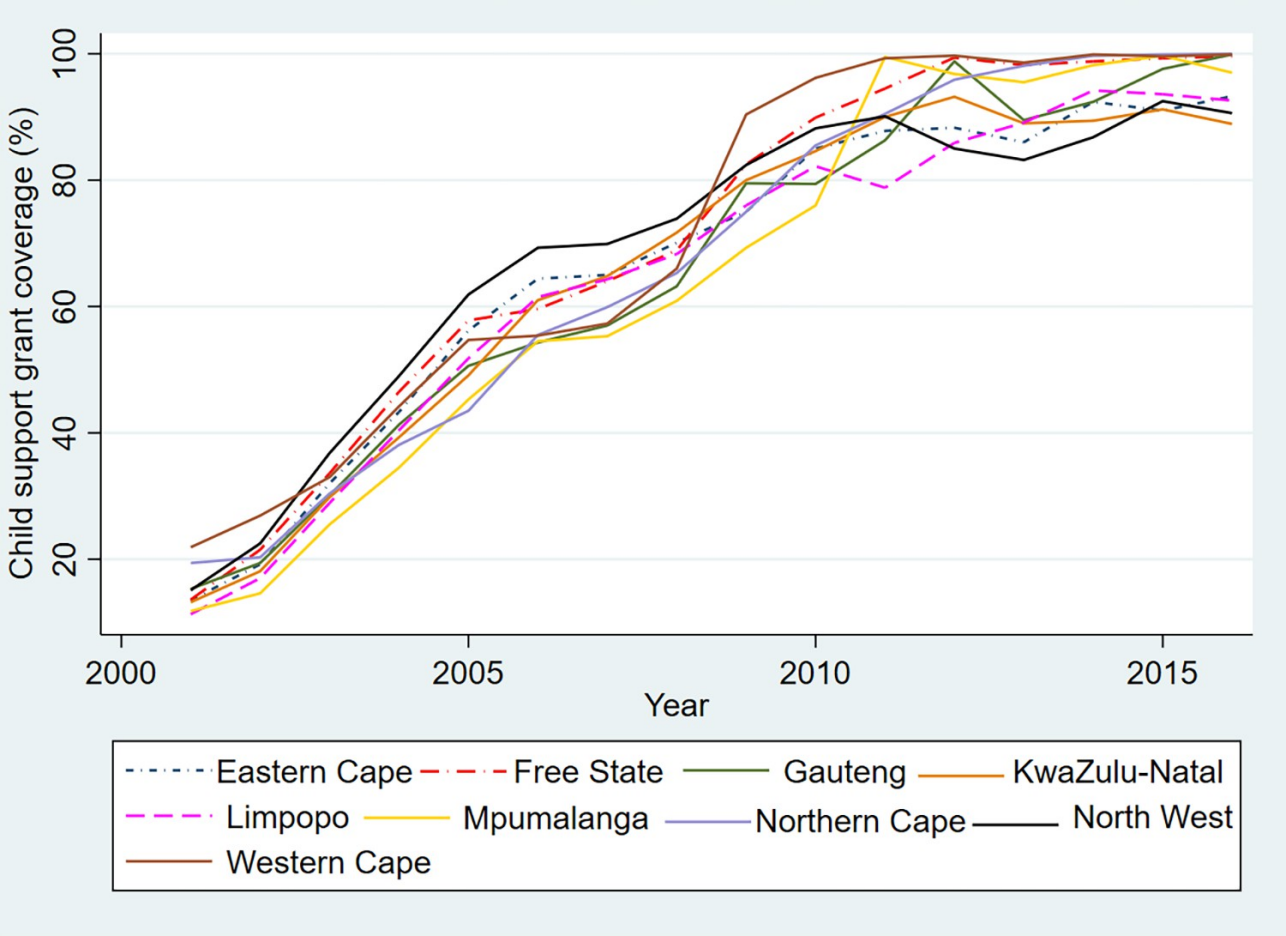
Provincial CSG coverage in South Africa 2001–2016. Source: Authors’ calculation using beneficiary data from SASSA *[27]* and population data from Stats SA *[33]*.

The CSG was introduced in 1998 with a starting amount of R100 per month (US$6.71 per month). It has become the largest social grant in the country accounting for 71% of all social grants disbursed in 2016 [31]. The CSG uptake has increased significantly over the years, from 1.9 million beneficiaries in 2001 to 12.1 million beneficiaries in 2016. The grant amount is increased to match inflation each year. The CSG is means tested, which means that only parents and caregivers whose income is below a certain level can get the grant. In 2016, a monthly grant of R420 (US$28.20 per month) was paid to parents and caregivers of children aged 0–17 years who earned an annual income of less than R48 600 (US$3 529) if single and R97 200 (US$7 058) if married [31]. CSG coverage is calculated by dividing the number of child support grant beneficiaries by the population of those aged 0–17 years old adjusted to include only the children living in poor households. Fig 4 shows trends in provincial CSG coverage.

## 3. Conceptual framework on the determinants of fertility

The primary focus of this study is to analyse the effect of CSG coverage and ART coverage variables within Becker’s [34] quality-quantity trade-off theoretical framework. In this framework, Angeles [2] argues that parental utility is a function of both child quantity and the education of each child (“child quality”). A trade-off between these two components arises since bringing up and educating children are both costly. Falling under-five mortality rates would thus allow a concentration on the “quality” of the children rather than a focus on increasing the number of children [2]. Furthermore, the intuition behind the quality-quantity model is that the income effect on child quantity may be negative, depending in part on the elasticity of substitution between child quality and quantity [35]. According to Burger et al. [10], educated mothers generally have higher earning potential and as such, the opportunity cost of raising a child is higher in terms of foregone salaries and wages. In other words, fertility will be low when the opportunity cost of female time is high. Higher levels of education may also elevate a woman’s knowledge of family planning and use of contraceptives [10].

Although the conceptual framework outlined thus far allows us to understand fertility determinants at the macro level, it must be stressed that this is purely an economic viewpoint. Amarante [35] points out that fertility is not only a rational response to changes in economic conditions, but that social interactions are also relevant to understanding fertility. Concurring with this view, Werding [36] points out that although it is important to apply a pure economic approach to fertility, it is also useful to note that decisions around fertility have a biological element, and are strongly rooted in a wide social context. Furthermore, the classical demographic transition theory may not be applicable for South Africa over the period 2001–2016. According to Moultrie [37], the kind of demographic transition experienced by South Africa during one of the worst HIV epidemics globally is atypical. South Africa was already at an advanced stage of demographic transition at the start of the HIV/AIDS epidemic.

Within this context, the level of fertility in a given population is determined by both direct and indirect factors [38]. The direct factors are also referred to as proximate determinants and include marriage rates (age at first marriage), contraceptive use, induced abortion, duration of breastfeeding, and postpartum abstinence, among others [38, 39]. The indirect factors are socio-economic and cultural factors that include education, rural-urban residence, religion, housing, health, income, social security, etc. The theoretical framework of Bongaarts [39] emphasises the role of the direct factors, while the theoretical frameworks proposed by Becker and Barro [6] as well as Becker [34] emphasize the role of indirect factors. Becker and Barro [6] provide an economic perspective on fertility determinants and emphasise the effects of parent’s income and the cost of bringing up children. This paper considers the economic, epidemiological, and demographic factors to explain fertility transitions.

Kalemli-Ozcan [40] points out that the economic approach to fertility predicts that: (1) improvement in women’s education increases the cost of childbearing and leads to fertility decline; and (2) reduction in under-five mortality is associated with a decline in fertility. Economic development, through increases in GDP per capita and urbanisation, is expected to change views towards fertility control. This liberates women from a patriarchal culture in which they are expected to focus on raising children [2].

Several studies [13, 14, 41] have shown that HIV/AIDS reduces fertility, implying that women living with HIV tend to bear fewer children. According to Moultrie and Timaeus [42], women who are aware of their HIV positive status may be unwilling to have children. This is due to the associated risks such as mother-to-child transmission (MTCT) of HIV and pregnancy complications because of the weakened immune systems. On the other hand, provision of ART is expected to have the opposite effect. Several individual individual-level studies have found significant increases in fertility after initiating ART [21–24].

With regards to marriage as a proximate determinant of fertility, Kuhnt and Trappe [43] suggest that men and women in partnerships, either married or cohabiting, will be more inclined to childbearing than those who are single. Gender preferences can increase fertility since childbearing is likely to continue until the optimal number of boys or girls is achieved [10]. The sex ratio at birth may serve as a proxy for gender preferences. Barber [44] found that the sex ratio at birth was associated with an increase in fertility while Chipman and Morrison [45] demonstrated that local sex ratios at birth have an impact on female fertility schedules.

Both Becker and Barro [6] and Bongaarts [39] have emphasised the importance of social security as a determinant of fertility. However, Becker and Barro’s [6] theoretical model focused on social security benefits related to retirement and found that a persistent increase in social security benefits reduces fertility. Others have focused on evaluating the ‘perverse incentives’ for fertility associated with child support grants, as discussed in Moultrie and McGrath [17] and Ngubane and Maharaj [18]. In this framework, an increase in social security could lead to increases in fertility holding other influences constant.

The link between cash transfers in the form of a CSG and fertility is not explained by Becker and Barro’s [6] quantity–quality trade-off. As explained by Angeles [2], the economic thinking here considers children as “normal goods” in line with Becker [34] and therefore higher income would lead to an increase in fertility. Contrary to this theoretical view, several studies in South Africa have pointed out that the CSG is not big enough to serve as an incentive to increase the size of the family but may be sufficient to encourage change towards lower fertility [15–18]. According to this thinking, the CSG income provides women with more control to decide their sexual and reproductive needs and facilitates the use of contraceptives through improvement in access to health services [46].

## 4. Literature review: Effects of under-five mortality, cash transfers and antiretroviral treatment

### 4.1 Under-five mortality and fertility

There are several theoretical and empirical studies that show that reductions in under-five mortality lead to declines in fertility [2, 3, 5–9, 35, 47]. Schultz [9] considers under-five mortality, income, employment, education, religion, nutrition, and family planning to be the main determinants of fertility. Furthermore, norms and values regarding family size and gender preference will also influence how under-five mortality affects fertility [48]. When under-five mortality is high, there is a tendency to have more children as insurance against the anticipated child losses [47]. A study by Amarante [35] found that under-five mortality is significantly associated with fertility in Latin America over the period 1960–2000. During the demographic transition, parents chose quality (education) over quantity (higher fertility), having fewer children and investing more in the education of each child [40, 49, 50].

Studies by Duflo [11] and Hansen & Lønstrup [12] found that fertility decreases with income and education, but they did not find any significant effect of under-five mortality on fertility. Along the same lines, analysis by Burger et al. [10] attributes most of the fertility decline in South Africa to improving levels of education and the declining prevalence of marriage. Although the study by Burger et al. [10] accounted for under-five mortality in their analysis, their study did not account for other important variables. For example, their study did not account for the CSG, HIV/AIDS and contraception prevalence.

### 4.2 Cash transfers and fertility

Few studies have accounted for social security protection for children when analysing determinants of fertility. Most of these studies [51–54] have investigated the impact of child focused cash transfers on fertility in the United States (US). The positive fertility impact of cash transfers found in these studies differs from the lack of such impact in developing countries [19]. Bastagli et al. [55] attribute the differences in the effect of cash transfers in developed and developing countries to the differences in the way the cash transfer programme is designed and the nature of the study.

In South Africa, few studies have evaluated the relationship between the CSG and teenage fertility, and these find no evidence of CSG impact on teenage fertility [15–18]. Using data from the 2007 Community Survey and the 2010 General Household Survey, Udjo [15] found that teenagers who receive the CSG are less likely to be pregnant with another child compared to those who do not receive the CSG. Based on the teenage fertility rates reported in the 1996 Census, 1998 Demographic Health Survey and 2001 Census, Moultrie and McGrath [17] suggested that it is unlikely that the CSG has led to an increase in teenage fertility rate given the observed decline of about 10 per cent in teenage fertility over this period. Using in-depth interviews with CSG recipients aged 18 to 24 years living in the northern KwaZulu-Natal in South Africa, Ngubane and Maharaj [18] similarly concluded that these young women did not fall pregnant to access the CSG.

Contrary to these studies, a recent study by Kollamparimbil [19] using the propensity score matching approach, found that although the CSG does not have a significant impact on fertility among teenage mothers, older mothers aged 20–58 years and who are CSG recipient have more children compared to those who do not receive the grant.

Although these studies allowed for the effect of social security transfers when assessing determinants of fertility, they suffer from methodological weaknesses. Apart from Kollamparambil [19], the focus of these studies was on fertility in teenagers and young women below 25 years, which is only one component of overall fertility. The study by Kollamparambil [19] did not control for other key determinants of fertility that are well established in the literature, and did not account for the effect of HIV/AIDS, ART and falling under-five mortality. Lastly, Kollamparambil [19] used propensity score matching for causal inference, which, according to King and Nielsen [20], may increase data imbalance, inefficiency, model dependence and bias.

Makiwane [16] used trend analysis covering an early period of 1998 to 2005 to examine data on teenage fertility and the uptake of the CSG in South Africa. This study found that the CSG did not have a significant association with teenage fertility. However, the study did not account for other determinants of fertility and did not use econometric techniques to evaluate the relationships. As in the study of Moultrie and McGrath [17], the period considered covered the early stages in the implementation of the CSG in South Africa, when the uptake was relatively low. Further methodological weaknesses of most of these studies are discussed by Udjo [15] and Kollamparimbil [19].

Although Udjo [15] applied logistic regression in his analysis of the effect of CSG on teenage fertility, the study relied on cross-sectional data from the 2007 Community Survey and the 2010 General Household Survey. Furthermore, the study by Udjo [15] did not control for under-five mortality, which potentially leads to omitted variable bias.

### 4.3 ART coverage and fertility

To the best of our knowledge, there has been no study thus far that has controlled for ART coverage when evaluating the determinants of fertility in South Africa. However, several epidemiological studies have found significant increases in fertility after starting ART [21–24]. In a recent study using routine data from health facilities in the Western Cape province of South Africa, Johnson et al. [21] found that receipt of ART is associated with high rates of second pregnancy among women who have recently been pregnant. A recent systematic review and meta-analysis by Yan et al. [56] found a strong association between being on ART and fertility desire, among people living with HIV. Therefore, a *priori* expectation is that ART rollout will lead to an increase in fertility.

## 5. Methodology

The aim of this paper is to examine how under-five mortality, the CSG and the provision of ART individually affect fertility in South Africa, while accounting for other determinants of fertility that are well established in the literature. In this paper, other direct and indirect determinants of fertility are controlled by HIV prevalence, real GDP per capita, mean years of schooling, marriage prevalence, contraception prevalence, urban ratio, and sex ratio at birth. The selection of independent variables is informed by the frameworks proposed by Becker and Barro [6], Becker [34] and Bongaarts [39]. In line with the approach by Amarante [35] and Kalemli-Ozcan [40], the baseline econometric specification of the total fertility rate is as follows:

$${TFR}_{it}=\alpha {U5\_MR}_{it}+\psi {CSG}_{it}+\gamma {ART}_{it}+{X\prime }_{it}\theta +\mu_{i}+\lambda_{t}+\varepsilon_{it}$$ (1)

where *TFR*_*it*_ is the total fertility rate for the province *i* at time t, *U*5_*MR*_*it*_ is the under-five mortality rate, *CSG*_*it*_ is the CSG coverage, *ART*_*it*_ is the ART coverage, *X*′_*it*_ is a vector of other control variables (HIV prevalence, real GDP per capita, education, marriage prevalence, contraception prevalence, urbanisation and sex ratio at birth), *μ*_*i*_ is the time-invariant province-specific fixed effects, *λ*_*t*_ is the time-variant fixed effects, and *ε*_*it*_ is a random error term.

The main empirical challenges when evaluating the impact of under-five mortality on fertility are the reverse causality and omitted variable biases [2, 40]. Specifically, higher levels of fertility may lead to higher under-five mortality, while unobservable factors may influence both under-five mortality and fertility. A recent South African study evaluating the impact of fertility choices on child health by Ohonba et al. [57] found that fertility choices are significantly associated with child health. Unobservable factors that may affect fertility are factors such as cultural beliefs, religion, and attitude to sexual relations. The 2SLS-FE-IV model used in this paper controls for the unobserved time-invariant differences between the individuals to ensure that the estimated coefficients are not biased due to omitted time-invariant characteristics [25]. Amarante [35] adds that it is reasonable to assume that part of the decline in under-five mortality is due to other economic variables that also affect the fertility decisions and that this would imply that estimation assuming exogeneity of under-five mortality would be biased. Schultz [9] found that the under-five mortality variable is endogenous in the fertility equation.

To deal with the endogeneity problem of the under-five mortality rate, this study employs the 2SLS-FE-IV approach using the xtivreg2 estimator in Stata to evaluate the impact of under-five mortality, CSG and ART coverage on fertility in South Africa. The xtivreg2 estimator implements instrumental variable /generalised method of moments (GMM) estimation of the fixed-effects and first-differences panel data models with possibly endogenous regressors [25]. An instrumental variable is a variable that does not itself explain changes in the dependent variable but is correlated with the endogenous explanatory variables and it is used in regression analysis when there are endogenous variables [25]. The estimation approach adopted in this paper is similar to the one adopted by Amarante [35].

Although our study adopts the 2SLS-FE-IV approach, results of the pooled ordinary least squares (pooled OLS), Random Effects (RE), and Fixed Effects (FE) models are also reported as a robustness check. Pooled OLS is the simplest estimation method used for panel data but does not recognise the panel structure of the data leading to biased estimates due to the presence of time invariant fixed effects [58]. Contrary to the RE model, the FE model has the advantage of not requiring the strict exogeneity condition which is often difficult to justify [25]. In the pooled OLS, RE and FE models, the error term is clustered at the province level to ensure that our results are robust against autocorrelation. Using 2SLS-FE-IV technique, the following baseline model is estimated in this paper.

$${TFR}_{it}=\beta_{0}+\beta_{1}{lnU5\_mr}_{it}+\beta_{2}{lnCSG}_{it}+\beta_{3}{lnART}_{it}+\beta_{4}{Educ}_{it}+\beta_{5}ln{RGDP\_pc}_{it}++\beta_{6}{lnHIV\_Prev}_{it}+\beta_{7}{CMR}_{it}+\beta_{8}{lnContraceptive}_{it}+\beta_{9}ln{Urban\_ratio}_{it}{+\beta_{10}{SRB}_{it}+\varepsilon }_{it}$$ (2)

where *TFR*_*it*_ is the total fertility rate, *lnU*5_*mr*_*it*_ is the natural log of under-five mortality rate, *lnCSG*_*it*_ is natural log of the CSG coverage, *lnART*_*it*_ is the natural log of ART coverage, *Educ*_*it*_ is the education proxied by mean years of schooling, *lnRGDP*_*pc*_*it*_ is the natural log of real GDP per capita, *lnHIV*_*Prev*_*it*_ is the natural log of HIV/AIDS prevalence, *CMR*_*it*_ represents marriage prevalence per thousand of the population, *lnContraceptive*_*it*_ is the natural log of contraception prevalence, *lnUrban*_*ratio*_*it*_ is the natural log of urban ratio which is a proxy for level of urbanisation and *SRB*_*it*_ is the sex ratio at birth.

Following the approach of Kalemli-Ozcan [40], Aksan [47] and Oster [59], the indicators of under-five mortality and real GDP per capita are on the natural logarithmic scale. Along the same lines, this study uses natural logs of indicators such as CSG coverage, ART coverage, HIV/AIDS prevalence and urban ratio to enable interpretation of results as elasticities. Kalemli-Ozcan [40] advises that although using the logs of percentage related variables makes the interpretation of regression results harder, it has several econometric advantages such as minimising the influence of outliers and making the estimated coefficients less prone to the scale effect due to factors such as underreporting. In order to account for variables that may have a non-linear relationship with the total fertility rate, we extend our model in Eq 3 to include the squared terms for indicators such as marriage prevalence rate, contraception prevalence and sex ratio at birth.


$${TFR}_{it}=\beta_{0}+\beta_{1}{lnU5_{mr}}_{it}+\beta_{2}{lnCSG}_{it}+\beta_{3}{lnART}_{it}+\beta_{4}{Educ}_{it}+\beta_{5}ln{RGDP_{pc}}_{it}+\beta_{6}{lnHIV_{Prev}}_{it}+\beta_{7}{CMR}_{it}+\beta_{8}{CMR}_{it}^{2}+\beta_{9}{lnContraceptive}_{it}+\beta_{10}{lnContraceptive}_{it}^{2}+\beta_{11}ln{Urban\_ratio}_{it}+\beta_{12}{SRB}_{it}+\beta_{13}{SRB}_{it}^{2}+\varepsilon_{it}$$ (3)

In this study, the under-five mortality is assumed to be endogenous, in line with the previous empirical studies [2, 9, 35]. Under-five mortality is mostly attributable to infectious diseases, poor quality of antenatal care, and lack of access to primary health care [35] and other factors such as malnutrition and poverty. In an attempt to get the valid and relevant instruments of the under-five mortality rate, we examined data on various measures of poverty like child hunger, child poverty, access to water, etc. However, these measures failed the validity and relevance tests during diagnostic checks. The under-five mortality rate is instead instrumented by the mother-to-child transmission rate of HIV and immunisation coverage. As noted at the start of section 2, declines in under-five mortality in recent decades have been attributed to the success of PMTCT programmes and increased vaccination [28–30]. This attempt to address the endogeneity problem through 2SLS FE IV regression follows the same approach as the one used in Schultz [9] and Amarante [35].

## 6. Data and variables

**Fig 5:**
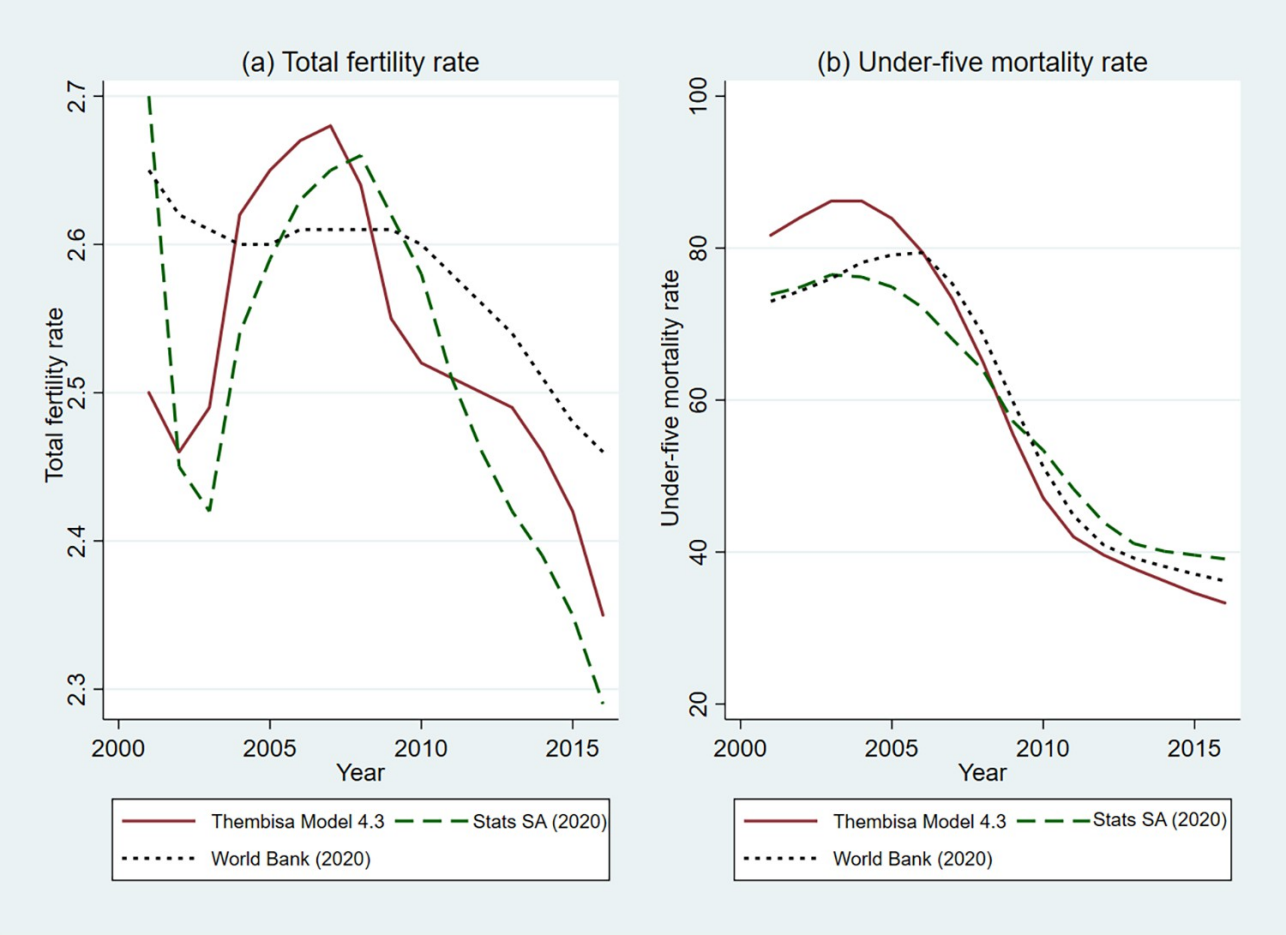
Comparison of TFR and under-five mortality rate estimates for South Africa. Source: Authors’ calculation using data from Johnson and Dorrington [26], Stats SA [61] and World Bank [62].

For the dependent variable, this study uses, consistent with the previous empirical literature [2, 35, 47], the total fertility rate as a measure of fertility. The provincial level TFR data are sourced from the Thembisa model, version 4.3 [26]. This is a combined demographic and HIV model, developed for South Africa. The model is calibrated to province-specific HIV prevalence data from antenatal clinic surveys and household surveys and is calibrated to be consistent with province-specific District Health Information System data on the numbers of patients receiving ART and uptake of HIV testing during pregnancy [60]. This process involves modelling under-five mortality rates using assumed non-HIV mortality rates, rates of mother-to-child transmission, rates of paediatric HIV disease progression and mortality, and province-specific data on access to paediatric ART [28]. Estimates of fertility at a provincial level are derived from several data sources: numbers of registered births and numbers of births recorded by the District Health Information System, censuses and Community Surveys, and school enrolment data [60]. Estimates of non-HIV under-five mortality rates are derived to be consistent with the registered deaths and deaths reported by households in the censuses and surveys. In this study, under-five mortality refers to the deaths between birth and the child’s fifth birthday. Comparison of estimates of the TFR and under-five mortality rate from various data sources shows that Thembisa model estimates are generally consistent with the estimates from Stats SA [61] and World Bank [62], as shown in Fig 5.

Data on under-five mortality rates, total HIV prevalence, HIV prevalence in females aged 15–49 years, MTCT of HIV, ART coverage and ART coverage in females aged 15–49 years are sourced from the Thembisa model, version 4.3 [26]. The CSG coverage is estimated by dividing the number of CSG beneficiaries by the population aged 0–17 years adjusted to include only the children living in households that are income-poor. The CSG beneficiary data is sourced from SASSA [27] while data on the population of children aged 0–17 years is sourced from Stats SA [33], excluding the children whose parents or caregivers are above the means test threshold.

Data on the education variable, which is proxied by the average (mean) years of schooling and the income variable (Real GDP per capita) are sourced from the Subnational Human Development Database [63]. The mean years of schooling refers to the average number of completed years of education in the population aged 25 years and older. The level of urbanisation is measured by the urban ratio which is the proportion of the population living in urban areas. Data on marriage prevalence (crude marriage prevalence rate) and urban ratios are sourced from Stats SA’s [33] annual General Household Surveys. Data on the sex ratio at birth are sourced from Stats SA’s [33] annual Recorded Live Births reports. Data on contraception prevalence, for which the couple-year protection rate is used as a proxy, and immunisation coverage are sourced from the Health Systems Trust’s [64] health indicators database. A detailed description of the data on dependent and explanatory variables is provided in S1 Table.

Owing to data limitations on key variables prior to 2001 and after 2016, the study covers only the period of 16 years from 2001 to 2016. Since there are 9 provinces, the study has a balanced panel of 144 observations.

## 7. Results

### 7.1 Descriptive statistics and scatter plots

Table 1 provides descriptive statistics of the dependant variable, regressors and instrumental variables.

**Table 1:** Descriptive statistics.

Variables	Abbreviated name	Obs	Mean	Std.Dev.	Min		Max
**Dependant variable**							
Total fertility rate	TFR	144	2.66	0.43		1.85	3.60
**Regressors**							
Under-five mortality rate (per 1 000 of the population)	U5_MR	144	58.8	25.8		17.1	112.1
CSG coverage (%)	CSG	144	46.7	19.4		8.8	75.6
ART coverage (%)	ART	144	17.6	17.7		0.15	60.2
ART coverage for females aged 15–49 years (%)	ART_15–49	144	18.5	18.6		0.2	62.6
Education (average years of schooling)	Educ_ays	144	9.0	1.3		5.8	11.9
Real GDP per capita (PPP 2011 US $)	RGDP_pc	144	11 628	3 814		5 393	19 620
Total HIV/AIDS prevalence (%)	HIV/AIDS_Prev	144	10.9	3.5		3.6	18.0
HIV/AIDS prevalence for females aged 15–49 years (%)	HIV_15–49	144	20.8	6.3		6.8	32.6
Marriage prevalence/ Crude marriage prevalence rate (per 1000 of the population)	CMR	144	274.2	51.6		198.2	363.3
Contraception prevalence (%)	Contraceptive	144	38.4	17.5		15.2	88.7
Urban ratio (%)/Urbanisation	Urban_ratio	144	59.0	26.8		10.6	98.2
Sex ratio at birth	SRB	144	101.4	1.07		98.1	104.2
**Instrumental variables**							
Immunisation coverage (%)	Immunisation	144	76.2	10.8		54.3	99.0
Mother to child transmission rate of HIV (%)	MTCT_HIV	144	18.9	12.0		4.3	43.9

**Fig 6:**
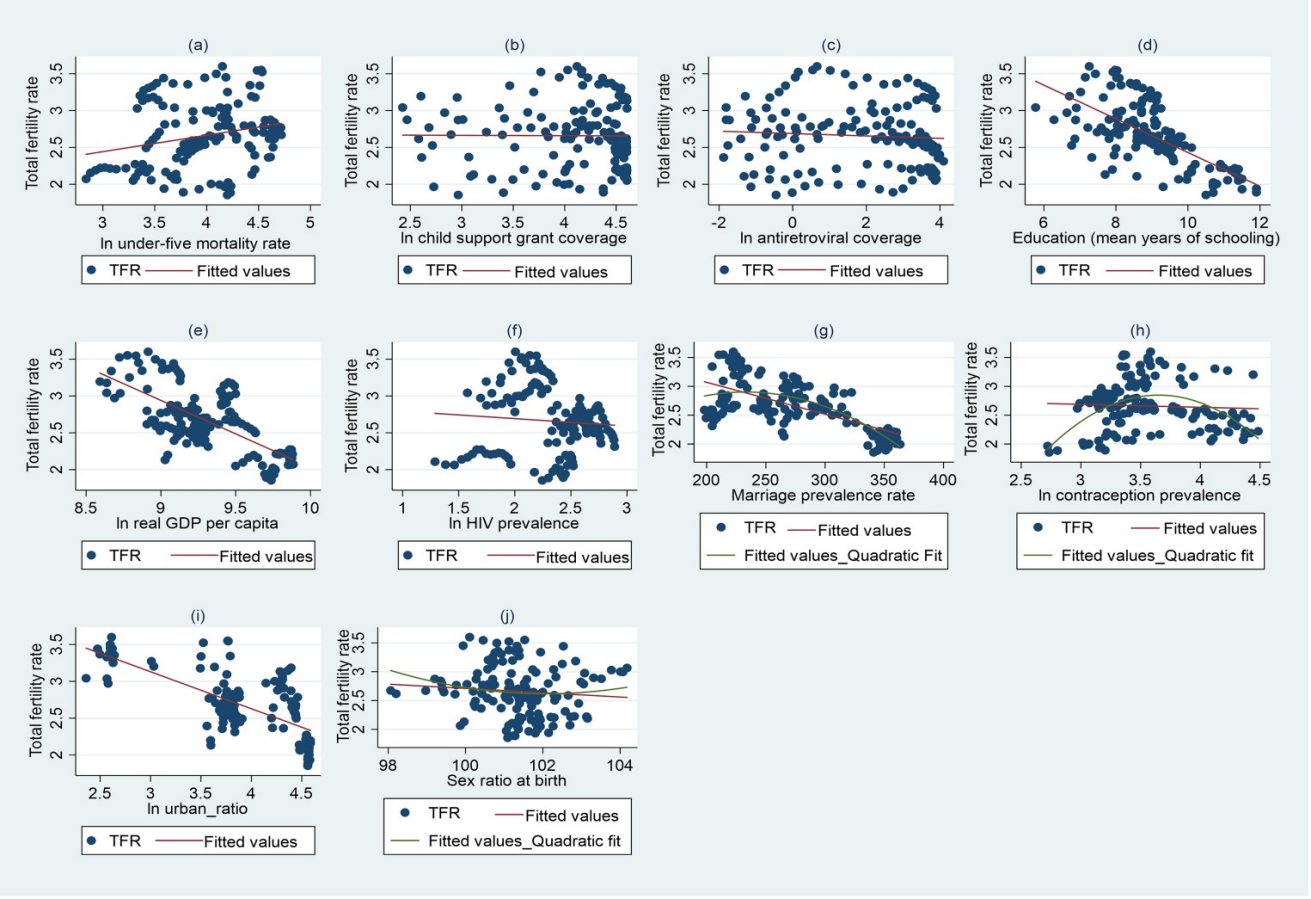
Scatter plots of total fertility rate and independent variables.

Table 1 shows that TFR varies from 1.85 to 3.60 children with a mean of 2.66 children. CSG coverage varies from 8.8% to 76.6% over the period 2001 to 2016. ART coverage varies from 0.2% to 60.2% with a mean of 17.6%. The remaining independent variables also show extensive variation over time across provinces. Fig 6 shows scatter plots of the TFR and independent variables, demonstrating the nature and strength of the bi-variate relationships.

Fig 6(A) shows that without controlling for any other variable, there exists a weakly positive relationship between the natural log of under-five mortality rate and the total fertility rate. On the other hand, there seem to be no discernible positive or negative relationship between the natural log of the CSG coverage, the natural log of ART coverage and the total fertility rate. The relationship between the natural log of HIV/AIDS prevalence and the total fertility rate is weakly negative and somewhat non-linear. A strong negative relationship is observed between the total fertility rate and the natural log of real GDP per capita, education and the natural log of the urban ratio. In the results section, we show that most of these relationships in the scatter plots are robust and do not reflect spurious correlations.

### 7.2 Correlation matrix of the explanatory variables

Before investigating the empirical impact of under-five mortality, CSG and ART on fertility, it is important to analyse the pairwise correlation between explanatory variables used in the regression. This helps to check if the results of the regression are affected by the presence of high levels of multicollinearity or not [65]. Results of the correlation matrix on the explanatory variables are presented in Table 2.

**Table 2:** Correlation matrix of explanatory variables used in the regression models.

	U5_MR	CSG	ART	RGDP_pc	Educ_ays	HIV_Prev	CMR	Contraceptive	Urban_ratio	SRB
U5_MR	1.000									
CSG	-0.688	1.000								
ART	-0.719	0.833	1.000							
RGDP_pc	-0.494	0.188	0.165	1.000						
Educ_Ays	-0.614	0.561	0.499	0.773	1.000					
HIV_Prev	0.209	0.723	0.342	-0.283	0.073	1.000				
CMR	-0.354	0.007	0.012	0.880	0.664	-0.348	1.000			
Contraceptive	-0.644	0.620	0.801	0.264	0.421	0.017	0.101	1.000		
Urban_Ratio	-0.291	0.127	0.103	0.882	0.721	-0.179	0.854	0.120	1.000	
SRB	-0.452	0.341	0.304	0.375	0.347	-0.235	0.344	0.298	0.372	1.000

From Table 2, there are several variables with high correlation coefficients, but no two explanatory variables have a correlation coefficient greater than 0.90. Sharma [65] points out that the need to reduce multicollinearity depends on its severity and the main goal of the regression analysis. Multicollinearity is particularly a problem if it is present in the independent variables of interest [66]. Most of the explanatory variables have correlation coefficients below 0.50. These bivariate correlations suggest that there is no evidence of near-perfect multicollinearity and as such, multicollinearity should not be a problem for the estimated regression models. However, the high correlation (0.83) between the CSG and ART variables is potentially concerning. This correlation emerges because both ART and the CSG were scaled up substantially over the same period (2001–2016), i.e., the correlation is serial or temporal in nature. As indicated earlier, the error term in the regression analysis is clustered at the province level to ensure that our results are fully robust against serial correlation. The Wooldridge [67] test statistic for autocorrelation in our baseline model in Eq 2 is 27.399 with a p-value of 0.001 which means that the null hypothesis of no first order autocorrelation is rejected. Hence we use Newey and West [68] standard errors that are fully robust against serial correlation. We also observe a high correlation of 0.80 between contraception prevalence and ART coverage. We demonstrate later that these correlations do not affect the precision of our coefficient estimates in the regression models.

### 7.3 Baseline estimates of the effect of under-five mortality, CSG and ART coverage on fertility

Table 3 contains the estimated effects of under-five mortality rate, CSG and ART coverage on fertility in South Africa as outlined in Eq 2. As mentioned earlier, four different alternative models are reported to check for robustness of our estimates.

**Table 3:** Baseline regression results: the effect of under-five mortality rate, CSG and ART on TFR.

Independent variables	Pooled OLS	RE	FE	2SLS-FE-IV
lnUnder-five mortality rate	0.714***(0.178)	0.714***(0.178)	0.548**(0.222)	0.635***(0.152)
lnCSG coverage	0.106(0.143)	0.106(0.143)	0.128(0.166)	0.099(0.105)
lnART coverage	0.325***(0.079)	0.325***(0.079)	0.239***(0.066)	0.266***(0.058)
Education	-0.301***(0.046)	-0.301***(0.046)	-0.197***(0.035)	-0.205***(0.053)
lnReal GDP per capita	-0.362*(0.175)	-0.362**(0.175)	-0.605(0.348)	-0.616***(0.178)
lnHIV/AIDS prevalence	-0.884***(0.131)	-0.884***(0.131)	-0.658(0.659)	-0.671**(0.280)
Crude marriage prevalence rate	0.003*(0.002)	0.003**(0.002)	0.004(0.003)	0.004***(0.002)
lnContraception prevalence	-0.167**(0.062)	-0.167***(0.062)	-0.194(0.136)	-0.180***(0.067)
lnUrban ratio	-0.143(0.097)	-0.143(0.097)	0.114(0.224)	0.104(0.249)
Sex ratio at birth	-0.012(0.020)	-0.012(0.020)	-0.014(0.019)	-0.015(0.014)
R^2^	0.84	0.84	0.39	0.39
Hansen J statistic				2.298(0.130)
Number of instruments				2
Endogeneity test				3.788(0.052)
Cragg-Donald Wald F statistic				276.875
Kleibergen-Paap rk LM statistic				42.115(0.000])
Hausman test		134.18(0.000)		

**Notes**: Robust standard errors (SEs) and p-values are given in parentheses. *, ** and ***denote significance at the 10%, 5% and 1% levels, respectively. MTCT rate of HIV and immunisation coverage are used as instruments for under-five mortality rate.

From Table 3, the pooled OLS and RE estimates are similar, suggesting that there are no random effects in the estimated equation. This observation is also supported by the Breusch and Pagan LM test for random effects, which is zero with a p-value of one. In this instance, the pooled OLS model is preferred over the RE model. The choice between the RE and FE models is based on the Hausman specification test [25]. Table 3 shows a Hausman test with a value of 134.18 with a p-value of zero, meaning that we reject the null hypothesis and hence the FE model is preferred over the RE model. This means that controlling for unobserved province-level fixed effects is important. As explained earlier, the under-five mortality rate variable is endogenous and as such, estimating the determinants of fertility using the FE model will lead to biased estimates. The endogeneity test has a chi-square value of 3.788 with a p-value of 0.052, meaning that the null hypothesis that under-five mortality is exogenous is rejected. The Hansen J statistic test for the validity of instruments has a value of 2.298 with a p-value of 0.130, which means that we cannot reject the null hypothesis that the MTCT rate of HIV and immunisation coverage are valid instruments for under-five mortality. The Cragg-Donald Wald F statistic is 276.875, which is significantly higher than the rule of thumb value of 10 and shows that the chosen instruments are not weak. This is also validated by the first stage F-statistics of 190.05 with a p-value of zero. The Kleibergen-Paap rk LM statistic test of 34.992 with a p-value of zero shows that the chosen instruments are relevant.

For our chosen 2SLS-FE-IV model in the last column of Table 3, most variables are statistically significant, with the expected signs in line with theory. The under-five mortality rate is statistically significant at the 1% level of significance. This result is robust to different methods of estimation since it is significant in all the models presented in Table 3.

As mentioned earlier in this paper, the theoretical prediction of the effect of the CSG coverage on fertility is dependent on whether or not the grant is big enough to encourage family expansion. In this paper, we find no significant relationship between the CSG coverage and the TFR in South Africa over the period 2001 to 2016.

ART coverage is statistically significant in all regression models in Table 3. Consistent with epidemiological studies [21–24], we find that an increase in the ART coverage is associated with an increase in the total fertility rate in South Africa. This result suggests that the elasticity of the TFR with respect to ART coverage is 0.27 in South Africa.

Except for the urban ratio and sex ratio at birth, coefficients of the control variables have the expected signs and are statistically significant. Consistent with the results of Burger et al. [10], Hansen and Lønstrup [12] and Aksan [47], we find that an increase in the average years of schooling (education) is associated with a decline in the TFR. This result reflects the higher opportunity cost of raising children in more highly educated women. In accordance with the results by Amarante [35], higher aggregate income (real GDP per capita) is associated with a decrease in the TFR in South Africa. HIV/AIDS prevalence is associated with fewer births and this finding is in line with results by Okada [13] and Young [14].

Lower marriage prevalence rates are associated with lower fertility, consistent with the South African study by Burger et al. [10]. The increased use of contraceptives is associated with lower total fertility rates in South Africa. Our results are robust to the use of alternative measures of HIV prevalence and ART coverage. In S2 Table we present results of Eq 2 using HIV prevalence data for females aged 15–49 years and ART coverage data for females aged 15–49 years.

### 7.4 Robustness checks

Hoechle [69] points out that although most empirical studies provide estimates with standard errors that are consistent in the presence of heteroscedasticity and serial correlation, cross-sectional dependence is still largely neglected. In our case, the CSG and ART rollout policies are formulated and managed by the central (national) government, which may lead to cross-sectional dependence within the provincial level panel data. The Breusch-Pagan LM test statistic of 100.368 with a p-value of 0 shows that the panel data used in this study exhibits some level of cross-sectional dependence. We do not perform Pesaran’s [70] cross-sectional dependence (CD) test since the CD test is appropriate for panels with N and T tending to infinity in any order [69]. In our case, both N and T are relatively small (9 and 16) and in our view, the CD test can be misleading. To perform robustness checks, we employ the Driscoll and Kraay [71] estimator that controls for cross-sectional dependence. The Driscoll and Kraay estimator produces standard errors that are consistent to heteroscedasticity and are robust to temporal and cross-sectional dependence [69]. However, the Driscoll and Kraay estimator assumes parameter exogeneity and as such, the 2SLS-FE-IV remains our preferred model. To account for potential endogeneity of some of the regressors (CSG and ART), we also provide results of the two-stage instrumental variables estimator (2SIV) for large-T panel data models, as developed by Norkute et al. [72]. The 2SIV estimator uses defactored covariates as instruments and provides a flexible specification of instruments [73]. When estimating the 2SIV model, we use the mean group (MG) estimator that considers potential heterogeneity of slope coefficients. Table 4 reports the pooled OLS, RE and FE estimates of the effect of under-five mortality, CSG and ART on TFR using Driscoll and Kraay standard errors. In addition, Table 4 also reports 2SIV estimator results obtained using the xtivdfreg Stata command of Kripfganz and Sarafidis [73].

**Table 4:** Determinants of fertility using Driscoll and Kraay standard errors and 2SIV-MG estimator.

	Driscoll and Kraay standard errors			2SIV-MG estimator
Independent variables	Pooled OLS (1)	RE (2)	FE (3)	2SIV-MG (4)
lnUnder-five mortality rate	0.714***(0.143)	0.714***(0.143)	0.548***(0.124)	0.618**(0.296)
lnCSG coverage	0.106(0.099)	0.106(0.099)	0.128(0.111)	-0.026(0.317)
lnART coverage	0.325***(0.056)	0.325***(0.056)	0.239***(0.051)	0.098(0.091)
Education	-0.301***(0.029)	-0.301***(0.029)	-0.197***(0.040)	-0.118***(0.032)
lnReal GDP per capita	-0.362**(0.161)	-0.362**(0.161)	-0.605***(0.183)	0.845(1.156)
lnHIV/AIDS prevalence	-0.884***(0.096)	-0.884***(0.096)	-0.658**(0.223)	0.794(2.395)
Crude marriage prevalence rate	0.003***(0.001)	0.003***(0.001)	0.004**(0.003)	-0.003(0.004)
lnContraception prevalence	-0.167***(0.037)	-0.167***(0.037)	-0.194**(0.136)	0.036(0.090)
lnUrban ratio	-0.143*(0.079)	-0.143*(0.079)	0.114(0.212)	0.673(0.579)
Sex ratio at birth	-0.012(0.013)	-0.012(0.013)	-0.014(0.011)	-0.012(0.015)
R^2^	0.84	0.84	0.39	
Hansen J statistic				0.000(1.000)
Number of instruments				14
Number of factors				1
Adjusted Delta (Slope heterogeneity test)				2.876 (0.004)
Hausman test		134.18(0.000)		

**Notes:** SEs and p-values are given in parentheses. *, ** and ***denote significance at the 10%, 5% and 1% levels, respectively.

We observe from Table 4 that the regression results reported in column (1) to (3) are qualitatively and quantitatively similar to those of the pooled OLS, RE and FE models obtained using Newey and West’s [68] cluster robust standard errors reported earlier in Table 3. The only difference is that the Driscoll and Kraay standard errors are much lower than those of Newey-West. Results obtained using the Driscoll and Kraay estimator are also qualitatively similar to those of our preferred 2SLS-FE-IV in Table 3 obtained using White’s [74] robust standard errors, suggesting that our results are robust to an alternative estimation strategy. Results of the 2SIV-MG model in column (4) show that the estimated coefficients of under-five mortality rate, ART and education are qualitatively similar to those of our preferred 2SLS-FE-IV model. However, only the under-five mortality rate and education variables are statistically significant. The coefficients of the 2SIV-MG model in column (4) and those of the 2SLS-FE-IV model in Table 3 differ for most variables owing to potential bias in the 2SIV-MG model given that our T is relatively small.

As discussed earlier in this section, we have observed a high correlation between our key variables of interest, CSG and ART coverage. In addition, a high correlation was also observed between contraception prevalence and ART coverage. To demonstrate robustness of our estimated coefficients, we re-estimate the baseline Eq 2 excluding CSG coverage and contraception prevalence as explanatory variables. Table 5 shows estimates of the effect of under-five mortality and ART coverage on total fertility rate after excluding CSG coverage.

**Table 5:** The effect of under-five mortality and ART coverage on TFR excluding the CSG coverage.

Independent variables	Pooled OLS	RE	FE	2SLS-FE-IV
ln Under-five mortality rate	0.789***(0.207)	0.789***(0.176)	0.677*(0.332)	0.723***(0.144)
lnART coverage	0.374***(0.044)	0.374***(0.044)	0.296**(0.093)	0.308***(0.043)
Education	-0.304***(0.047)	-0.304***(0.047)	-0.194***(0.036)	-0.201***(0.053)
lnReal GDP per capita	-0.339*(0.169)	-0.339**(0.169)	-0.661*(0.351)	-0.656***(0.179)
lnHIV/AIDS prevalence	-0.925***(0.099)	-0.925***(0.099)	-0.592(0.765)	-0.615**(0.275)
Crude marriage prevalence rate	0.004**(0.002)	0.004**(0.002)	0.039(0.003)	0.004***(0.002)
lnContraception prevalence	-0.171**(0.058)	-0.171***(0.058)	-0.199(0.131)	-0.188***(0.067)
lnUrban ratio	-0.157(0.105)	-0.157(0.105)	0.128(0.213)	0.117(0.250)
Sex ratio at birth	-0.011(0.019)	-0.011(0.019)	-0.013(0.019)	-0.014(0.017)
R2	0.84	0.84	0.38	0.38
Hansen J statistic				1.615(0.204)
Number of instruments				2
Endogeneity test				3.762(0.049)
Cragg-Donald Wald F statistic				390.326
Kleibergen-Paap rk LM statistic				54.190(0.000)
Hausman test		2.93(0.967)		

Notes: Robust SEs and p-values are given in parentheses. *, ** and ***denote significance at the 10%, 5% and 1% levels, respectively. MTCT rate of HIV and immunisation coverage are used as instruments for under-five mortality rate.

Comparing regression results in Table 5 to those of our baseline model in Table 3, the effects of under-five mortality and ART and other control variables remain qualitatively similar in terms of the sign and level of significance. Furthermore, there is minimal change in the magnitude of the estimated coefficients. As such, there is no evidence that multicollinearity distorts the results of the regression models. Similar results are observed when the CSG coverage and contraception prevalence variables are excluded in the regression models (S3 Table). Overall, dropping these explanatory variables decreases the standard errors of most coefficients slightly without improving the explanatory power of the models as evidenced by a slight decline in R-squared in our chosen 2SLS-FE-IV model.

We also demonstrate that our results are not sensitive to the inclusion of squared terms on marriage prevalence rate, contraception prevalence and sex ratio at birth variables. Table 6 contains the estimated effects on the TFR when including the squared terms, as outlined in Eq 3.

**Table 6:** Determinants of TFR including squared terms for some control variables.

Independent variables	Pooled OLS	RE	FE	2SLS-FE-IV
ln Under-five mortality rate	0.743***(0.207)	0.743***(0.207)	0.484**(0.161)	0.569***(0.142)
lnCSG coverage	0.161(0.165)	0.161(0.165)	0.263(0.243)	0.230(0.127)
lnART coverage	0.314***(0.094)	0.314***(0.094)	0.231***(0.061)	0.257***(0.057)
Education	-0.243***(0.046)	-0.243***(0.046)	-0.266***(0.063)	-0.273***(0.050)
lnReal GDP per capita	-0.486*(0.220)	-0.486**(0.220)	-0.514(0.375)	-0.523***(0.193)
lnHIV/AIDS prevalence	-0.996***(0.163)	-0.996***(0.163)	-0.664(0.834)	-0.661**(0.335)
Marriage prevalence rate	0.017(0.010)	0.017(0.010)	0.039**(0.015)	0.039***(0.012)
Marriage prevalence rate squared	-0.00002(0.000)	-0.00002(0.000)	-0.00006**(0.000)	-0.00006***(0.000)
lnContraception prevalence	-1.945*(0.017)	-1.945**(0.017)	-2.137(1.456)	-2.064***(0.718)
lnContraception prevalence squared	0.241*(0.130)	0.241*(0.130)	0.263(0.205)	0.254***(0.097)
lnUrban ratio	-0.154(0.099)	-0.154(0.099)	-0.188(0.319)	-0.194(0.235)
Sex ratio at birth	-3.359**(1.134)	-3.359***(1.134)	-0.316(0.912)	-0.178(1.482)
Sex ratio at birth squared	0.016**(0.006)	0.016***(0.006)	0.002(0.004)	0.008(0.007)
R^2^	0.86	0.86	0.47	0.47
Hansen J statistic				1.174(0.556)
Number of instruments				2
Endogeneity test				4.762(0.029)
Cragg-Donald Wald F statistic				172.168
Kleibergen-Paap rk LM statistic				42.404(0.000)
Hausman test		49.90(0.000)		

Notes: Robust SEs and p-values are given in parentheses. *, ** and ***denote level of significance at 10%, 5% and 1%, respectively. MTCT rate of HIV and immunisation coverage are used as instruments for under-five mortality rate.

Statistical significance of the squared terms for the marriage prevalence and contraception prevalence variables suggests that their relationship with TFR may be non-linear. The negative sign on the squared marriage prevalence variable suggests that at low levels, marriage prevalence is associated with an increase in TFR, but for high levels, marriage prevalence is associated with a decline in TFR. The opposite is observed in the case of contraception prevalence.

In S4 Table, we present results of similar specification outlined in Eq 3 using HIV prevalence data for females aged 15–49 years and ART coverage data for females aged 15–49 years. The results in S4 Table are similar to those presented in Table 6. Further robustness checks were conducted by excluding variables such as urban ratio and sex ratio at birth. These estimates are presented in S5 Table. Overall, our results are robust to alternative estimation strategies, measures of HIV prevalence and ART coverage and model specification.

## 8. Discussion and conclusions

Determinants of fertility are frequently contested in the public discourse. In the African setting, there is little consensus on the effects of child support grants and expanding access to HIV treatment. This paper adds to the growing research to improve our knowledge of the determinants of fertility by investigating the effects of under-five mortality rate, CSG coverage and ART coverage on fertility in South Africa over the period 2001 to 2016. This period was characterised by significant increases in the CSG and ART coverage. Furthermore, this period was characterised by significant declines in the under-five mortality rate mainly due to PMTCT and ART. Despite epidemiological data showing the effect of ART on fertility at the individual level, no study has examined the effects of ART coverage on population-level fertility in the economic literature. Although there are a few studies in South Africa that have evaluated the effect of the CSG on teenage pregnancy, there are no studies that have assessed the effect on fertility over all ages.

Using provincial-level data from a panel of nine South African provinces over the period 2001–2016, we find no evidence to support the hypothesis that the increase in coverage of the CSG is associated with an increase in fertility. In other words, we find no evidence that there are perverse incentives to have a child associated with the child support grant. This finding is consistent with the empirical results of Udjo [15], Makiwane [16] and Moultrie and McGrath [17] who found no significant association between the CSG and teenage fertility in South Africa. Therefore, our results add to this developing literature by demonstrating that CSG is not associated with childbearing behaviour (in aggregate, over all ages). However, our results contrast with the finding by Kollamparimbil [19] who suggested that older mothers receiving the CSG have had more children compared to those that do not receive the grant. As we have argued earlier in this paper, the finding by Kollamparimbil [19] may be due to potential omitted variable bias and the bias induced by the propensity score matching technique. Another possible explanation for our finding is that some of the caregivers who receive the grant are not the biological parents of the children under their care and are unlikely to have children because they want to receive the grant [18].

On the other hand, our results indicate that an increase in the coverage of ART is associated with an increase in the total fertility rate, which could explain why the fertility decline in South Africa over the period 2001 to 2016 has been relatively modest when compared to that in earlier decades. This finding suggests that studies that evaluate the determinants of fertility in countries with significant HIV epidemics should account for ART coverage in their analysis. Failure to account for ART coverage can lead to omitted variable bias. This has policy implications for South Africa. Although the scale up of ART has improved health outcomes, the rollout should also be linked with further family planning initiatives to minimise unintended pregnancies. Several studies [75–77] confirm high incidences of unintended pregnancy in women who are HIV-positive and on treatment. Adeniyi et al. [78] advises that combining services such as HIV care and family planning among HIV-positive women is important to reduce unintended pregnancy. Furthermore, Homsy et al. [75] emphasise that there should be regular counselling for women who are on ART and their partners, on the fertility enhancing effects of ART. Health service providers should increase the provision of reproductive health services and sexuality education enabling people to decide freely on the desired size of the family through careful planning of pregnancies [22].

We have also found that a decline in under-five mortality is associated with a decline in fertility. The finding that a decrease in under-five mortality is associated with a decline in fertility is consistent with theoretical and empirical literature [2, 5, 35, 47]. The 2SLS-FE-IV estimates reported earlier in Table 3 suggest that the elasticity of the TFR with respect to the under-five mortality rate is 0.64 in South Africa over the period 2001 to 2016. The size of the effect that we find for South Africa is similar to the estimates of Angeles [2] and the ones reported by Aksan [47]. Elasticities of 0.67 for Middle East and North Africa, 0.68 for Latin America and the Caribbean and 0.71 in East Asia are reported in Aksan [47].

Education, real GDP per capita, HIV prevalence, marriage prevalence and contraception prevalence are also important determinants of fertility in South Africa. However, our analysis does not find a significant relationship between urbanisation or the sex ratio at birth, and fertility.

A limitation of this study is that we did not extend the data set beyond 2016 because variables such as the under-five mortality rate and to a lesser extent the TFR were less reliably estimated in recent periods. This is mainly due to the lack of a census or community survey since 2016. Also, due to lack of data on important variables like CSG and ART coverage, the study could not cover the period prior to 2001. As such, our study starts two years before the peak in under-five mortality due to HIV/AIDS. Thus, the study does not capture the increase in under-five mortality, due to HIV/AIDS, prior to 2001. Despite these limitations, the finding that the decline in under-five mortality is associated with a decline in fertility holds over the sample period. Future studies might address the related problem of small observation numbers by using municipal or district level data when these become available. In addition, future studies should also consider investigating the long-term relationship between under-five mortality rate and fertility in South Africa.

## Supporting information

S1 Table Data description. (DOCX)

S2 Table Determinants of fertility using HIV and ART data for females aged 15–49 years. Notes: Robust SEs and p-values are given in parentheses. *, ** and ***Denote significance at the 10%, 5% and 1% levels, respectively. MTCT rate of HIV and immunisation coverage are used as instruments for under-five mortality rate.
(DOCX)

S3 Table Determinants of fertility excluding CSG and contraception prevalence. Notes: Notes: Robust SEs and p-values are given in parentheses. *, ** and ***denote significance at the 10%, 5% and 1% levels, respectively. MTCT rate of HIV and Immunisation coverage are used as instruments for Under-five mortality rate.
(DOCX)

S4 Table Determinants of fertility including squared terms using HIV and ART data for females aged 15–49 years. Notes: Robust SEs and p-values are given in parentheses. *, ** and ***Denote significance at the 10%, 5% and 1% levels, respectively. MTCT rate of HIV, immunisation coverage and access to piped water are used as instruments for under-five mortality rate.
(DOCX)

S5 Table Determinants of fertility excluding urban ratio and sex ratio at birth. Notes: Robust SEs and p-values are given in parentheses. *, ** and ***denote significance at the 10%, 5% and 1% levels, respectively. MTCT rate of HIV and immunisation coverage are used as instruments for under-five mortality rate.
(DOCX)

S1 Dataset Panel data used in the econometric (regression) analysis. (XLSX)

S1 File Stata Do file used to generate econometric estimates. (DO)
