# Supplementary material for: The impact of child mortality on fertility in South Africa: Do child support grants and antiretroviral treatment matter?
Source: PLoS One. 2023 Apr 4;18(4):e0284032. doi: 10.1371/journal.pone.0284032 (PMC10072469; doi:10.1371/journal.pone.0284032)
Supplement: S1 Table — (DOCX) [file pone.0284032.s001.docx]

**S1 Table.** **Data description.**

| **Variable** | **Description** | **Source** |
| --- | --- | --- |
| Total fertility rate | TFR at a point in time is the average number of children women would have by the time they reach 49 years if the women experienced the age-specific fertility rates measured at that point in time (Johnson & Dorrington, 2020a). | Thembisa model version 4.3 (Johnson & Dorrington, 2020a) |
| Under-five mortality rate | The number of children who die under the age of 5 years per 1 000 live births (Johnson & Dorrington, 2020a). | Thembisa model version 4.3 (Johnson & Dorrington, 2020a) |
| Education (mean years of schooling) | Calculates the average number of years of completed education of a population aged 25 years and older, excluding years spent repeating individual grades. | The Subnational Human Development Database (Smits & Permanyer, 2021) |
| Real GDP per capita | This is a measure of the country’s total economic output divided by the number of people and adjusted for inflation (Smits & Permanyer, 2021). Data on GDP per capita is adjusted for purchasing power parity (PPP) based on the 2011 US dollars ($). | The Subnational Human Development Database (Smits & Permanyer, 2021) |
| Child support grant coverage | This is the percentage of children receiving the state cash transfers. It is estimated by dividing the number of CSG beneficiaries by the population of those aged 0-17 years old adjusted to include only the children living in poor households. In South Africa, the CSG is means tested and is available to qualifying children under 18 years. Government provides this social assistance to children from poor households and is paid to parents or caregivers of eligible children. In 2016, the income threshold to qualify for the grant was R4 200 (USD $301) per month for a single caregiver and if the caregiver is married, the threshold was R8 600 (USD $602) per month. (National Treasury, 2021). | The number of beneficiaries’ data are sourced from the South African Social Security Agency’s (2021) SOCPEN database while the population data are sourced from Stats SA’s (2020b) General Household Surveys. |
| Antiretroviral coverage | This is the number of individuals receiving antiretroviral treatment at a point in time divided by the number of individuals needing treatment (Johnson & Dorrington, 2020a). | Thembisa model version 4.3 (Johnson & Dorrington, 2020a) |
| Total HIV/AIDS prevalence rate | Refers to the percentage of people infected with Human Immunodeficiency Virus (HIV). | Thembisa model version 4.3 (Johnson & Dorrington, 2020a) |
| Marriage prevalence rate | In line with Johnson & Dorrington (2020), this paper defines married individuals as those who are legally married or living together with their main partner. The marriage prevalence rate in this paper is defined as the number of those ‘married’ per 1 000 of the population. Therefore, the calculation of marriage prevalence rate is on the prevalence of marriage at a point in time rather than recorded marriages per year and is a crude marriage rate since total population is used. | Annual General Household Surveys (Stats SA, 2020b). |
| **Variable** | **Description** | **Source** |
| Contraception prevalence | This is the percentage of women protected against pregnancy using latest contraception methods which include sterilisations as a proportion of female population aged 15-49 years and it is proxied by couple year protection (Health Systems Trust, 2020). Couple year protection is the total of (Oral pill cycles/15)+(Medroxyprogesterone injection/4)+(Norethisterone enanthate injection/6)+(Intrauterine contraceptive device X 4.5)+)+(Sub dermal implant X 2.5)+Male condoms distributed/120)+(Female condoms distributed/120)+(Male sterilisation X 10)+(Female sterilisation X 10). | Health Systems Trust (2020) |
| Urban ratio | The share of the population in percentage living in urban areas. In this paper, this includes both urban formal and urban informal | Annual General Household Surveys (Stats SA, 2020b). |
| Sex ratio at birth | This is defined as the ratio of male births to the female births in the population and it is scaled to a denominator of 100 (Stats SA, 2020b). | Annual Recorded Live Births Reports (Stats SA, 2020b) |
| Immunisation coverage | The percentage of children in the target area under one year who finish their primary course of immunisation (Health Systems Trust, 2020). | Health Systems Trust (2020) |
| Mother-to-child transmission of HIV | This is a transmission of HIV from a mother living with HIV to her child during pregnancy, labour, delivery or breastfeeding (Johnson & Dorrington, 2020a). | Thembisa model version 4.3 (Johnson & Dorrington, 2020a) |
