# Supplementary material for: The impact of child mortality on fertility in South Africa: Do child support grants and antiretroviral treatment matter?
Source: PLoS One. 2023 Apr 4;18(4):e0284032. doi: 10.1371/journal.pone.0284032 (PMC10072469; doi:10.1371/journal.pone.0284032)
Supplement: S2 Table — Notes: Robust SEs and p-values are given in parentheses. *, ** and ***Denote significance at the 10%, 5% and 1% levels, respectively. MTCT rate of HIV and immunisation coverage are used as instruments for under-five mortality rate. (DOCX) [file pone.0284032.s002.docx]

**S2 Table. Determinants of fertility using HIV and ART data for females aged 15-49 years.**

| **Independent variables** | **Pooled OLS** | **RE** | **FE** | **2SLS-FE-IV** |
| --- | --- | --- | --- | --- |
| lnUnder-five mortality rate | 0.817***(0.171) | 0.817***(0.171) | 0.548**(0.230) | 0.664***(0.155) |
| lnCSG coverage | 0.074(0.132) | 0.074(0.132) | 0.099(0.137) | 0.060(0.102) |
| lnART coverage for females aged 15-49 years | 0.359***(0.070) | 0.359***(0.070) | 0.246**(0.074) | 0.283***(0.061) |
| Education | -0.291***(0.033) | -0.291***(0.033) | -0.203***(0.033) | -0.213***(0.052) |
| lnReal GDP per capita | -0.486**(0.180) | -0.486***(0.180) | -0.716(0.426) | -0.727***(0.191) |
| lnHIV prevalence for females aged 15-49 years | -1.078***(0.147) | -1.078***(0.147) | -0.537(0.786) | -0.568**(0.275) |
| Marriage prevalence rate | 0.003*(0.002) | 0.003**(0.002) | 0.004(0.003) | 0.004***(0.002) |
| lnContraception prevalence | -0.110*(0.058) | -0.110*(0.058) | -0.204(0.134) | -0.186***(0.014) |
| lnUrban ratio | -0.160*(0.081) | -0.160**(0.081) | 0.102(0.232) | 0.091(0.254) |
| Sex ratio at birth | -0.018(0.020) | -0.018(0.020) | -0.014(0.019) | -0.016(0.017) |
| R^2^ | 0.85 | 0.85 | 0.37 | 0.37 |
| Hansen J statistic |  |  |  | 1.811(0.178) |
| Number of instruments |  |  |  | 2 |
| Endogeneity test |  |  |  | 3.830(0.050) |
| Cragg-Donald Wald F statistic |  |  |  | 272.893 |
| Kleibergen-paap rk LM statistic |  |  |  | 45.401(0.000) |
| Hausman |  | 56.40(0.000) |  |  |

**Notes:** Robust SEs and p-values are given in parentheses. *, ** and ***Denote significance at the 10%, 5% and 1% levels, respectively. MTCT rate of HIV and immunisation coverage are used as instruments for under-five mortality rate.
