# Supplementary material for: The impact of child mortality on fertility in South Africa: Do child support grants and antiretroviral treatment matter?
Source: PLoS One. 2023 Apr 4;18(4):e0284032. doi: 10.1371/journal.pone.0284032 (PMC10072469; doi:10.1371/journal.pone.0284032)
Supplement: S3 Table — Notes: Notes: Robust SEs and p-values are given in parentheses. *, ** and ***denote significance at the 10%, 5% and 1% levels, respectively. MTCT rate of HIV and Immunisation coverage are used as instruments for Under-five mortality rate. (DOCX) [file pone.0284032.s003.docx]

**S3 Table. Determinants of fertility excluding CSG and contraception prevalence**

| **Independent variables** | **Pooled OLS** | **RE** | **FE** | **2SLS-FE-IV** |
| --- | --- | --- | --- | --- |
| ln Under-five mortality rate | 0.868***(0.162) | 0.868***(0.162) | 0.860**(0.270) | 0.950***(0.126) |
| lnART coverage | 0.355***(0.050) | 0.355***(0.050) | 0.310**(0.098) | 0.337***(0.046) |
| Education | -0.293***(0.058) | -0.293***(0.058) | -0.204***(0.048) | -0.220***(0.057) |
| lnReal GDP per capita | -0.326(0.178) | -0.326*(0.178) | -0.543(0.444) | -0.547***(0.195) |
| lnHIV/AIDS prevalence | -0.904***(0.109) | -0.904***(0.109) | -0.712(0.767) | -0.750***(0.275) |
| Marriage prevalence rate | 0.004*(0.002) | 0.004**(0.002) | 0.004(0.003) | 0.004**(0.002) |
| lnUrban ratio | -0.167(0.113) | -0.167(0.113) | 0.025(0.235) | 0.014(0.256) |
| Sex ratio at birth | -0.010(0.023 | -0.010(0.023) | -0.022(0.019) | -0.023(0.018) |
| R2 | 0.83 | 0.83 | 0.34 | 0.34 |
| Hansen J statistic |  |  |  | 1.615(0.204) |
| Number of instruments |  |  |  | 2 |
| Endogeneity test |  |  |  | 6.678(0.009) |
| Cragg-Donald Wald F statistic |  |  |  | 466.446 |
| Kleibergen-Paap rk LM statistic |  |  |  | 46.647(0.000) |
| Hausman |  | 6.63(0.577) |  |  |

**Notes:** Robust SEs and p-values are given in parentheses. *, ** and ***denote significance at the 10%, 5% and 1% levels, respectively. MTCT rate of HIV and Immunisation coverage are used as instruments for Under-five mortality rate.
