# Supplementary material for: The impact of child mortality on fertility in South Africa: Do child support grants and antiretroviral treatment matter?
Source: PLoS One. 2023 Apr 4;18(4):e0284032. doi: 10.1371/journal.pone.0284032 (PMC10072469; doi:10.1371/journal.pone.0284032)
Supplement: S4 Table — Notes: Robust SEs and p-values are given in parentheses. *, ** and ***Denote significance at the 10%, 5% and 1% levels, respectively. MTCT rate of HIV, immunisation coverage and access to piped water are used as instruments for under-five mortality rate. (DOCX) [file pone.0284032.s004.docx]

**S4 Table. Determinants of fertility including squared terms using HIV and ART data for females aged 15-49 years**.

| **Independent variables** | **Pooled OLS** | **RE** | | **FE** | **2SLS-FE-IV** |
| --- | --- | --- | --- | --- | --- |
| lnUnder-five mortality rate | 0.855***(0.198) | | 0.855***(0.198) | 0.503**(0.168) | 0.607***(0.142) |
| lnCSG coverage | 0.136(0.156) | | 0.136(0.156) | 0.232(0.220) | 0.191(0.122) |
| lnART coverage for females aged 15-49 years | 0.352***(0.086) | | 0.352***(0.086) | 0.245***(0.066) | 0.2778***(0.059) |
| Education | -0.240***(0.044) | | -0.240***(0.044) | -0.277***(0.065) | -0.287***(0.050) |
| lnReal GDP per capita | -0.627**(0.229) | | -0.627***(0.229) | -0.598(0.431) | -0.608***(0.205) |
| lnHIV prevalence for females aged 15-49 years | -1.210***(0.182) | | -1.210***(0.182) | -0.574(0.791) | -0.583*(0.328) |
| Marriage prevalence rate | 0.016(0.010) | | 0.016(0.010) | 0.044**(0.017) | 0.044***(0.011) |
| Marriage prevalence rate squared | -0.00002(0.000) | | -0.00002(0.000) | -0.0001**(0.000) | -0.0001***(0.000) |
| lnContraception prevalence | -2.071*(0.960) | | -2.071**(0.960) | -2.031(1.475) | -1.952***(0.749) |
| lnContraception prevalence squared | 0.267*(0.131) | | 0.267**(0.131) | 0.248(0.207) | 0.239(0.101) |
| lnUrban ratio | -0.171(0.083) | | -0.171(0.083) | -0.211(0.312) | -0.218(0.231) |
| Sex ratio at birth | -2.976**(1.122) | | -2.976***(1.122) | -0.215(0.975) | -0.066(1.473) |
| Sex ratio at birth squared | 0.015**(0.006) | | 0.015***(0.006) | 0.0009(0.005) | -0.0002(0.007) |
| R^2^ | 0.86 | 0.86 | | 0.46 | 0.46 |
| Hansen J statistic |  |  | |  | 1.012(0.603) |
| Number of instruments |  |  | |  | 2 |
| Endogeneity test |  |  | |  | 3.152[0.076] |
| Cragg-Donald Wald F statistic |  |  | |  | 171.699 |
| Kleibergen-paap rk LM statistic |  |  | |  | 44.436[0.000] |
| Hausman test |  | 61.49[0.000] | |  |  |

**Notes:** Robust SEs and p-values are given in parentheses. *, ** and ***Denote significance at the 10%, 5% and 1% levels, respectively. MTCT rate of HIV, immunisation coverage and access to piped water are used as instruments for under-five mortality rate.
