# Supplementary material for: The impact of child mortality on fertility in South Africa: Do child support grants and antiretroviral treatment matter?
Source: PLoS One. 2023 Apr 4;18(4):e0284032. doi: 10.1371/journal.pone.0284032 (PMC10072469; doi:10.1371/journal.pone.0284032)
Supplement: S5 Table — Notes: Robust SEs and p-values are given in parentheses. *, ** and ***denote significance at the 10%, 5% and 1% levels, respectively. MTCT rate of HIV and immunisation coverage are used as instruments for under-five mortality rate. (DOCX) [file pone.0284032.s005.docx]

**S5 Table. Determinants of fertility excluding urban ratio and sex ratio at birth.**

| **Independent variables** | **Pooled OLS** | **RE** | **FE** | **2SLS-FE-IV** |
| --- | --- | --- | --- | --- |
| lnUnder-five mortality rate | 0.563**(0.188) | 0.563***(0.188) | 0.545**(0.226) | 0.628***(0.148) |
| lnCSG coverage | 0.161(0.182) | 0.161(0.182) | 0.128(0.165) | 0.099(0.106) |
| lnART coverage | 0.280**(0.097) | 0.280***(0.097) | 0.239**(0.078) | 0.264***(0.058) |
| Education | -0.306***(0.036) | -0.306***(0.036) | -0.203***(0.038) | -0.209***(0.049) |
| lnReal GDP per capita | -0.524***(0.119) | -0.524***(0.119) | -0.581(0.326) | -0.591***(0.154) |
| lnHIV/AIDS prevalence | -0.833***(0.148) | -0.833***(0.148) | -0.638*(0.816) | -0.651**(0.274) |
| Marriage prevalence rate | 0.003(0.002) | 0.003*(0.002) | 0.004(0.003) | 0.004***(0.001) |
| lnContraception prevalence | -0.175***(0.051) | -0.175***(0.051) | -0.196(0.124) | -0.183***(0.063) |
| R^2^ | 0.83 | 0.83 | 0.38 | 0.38 |
| Hansen J statistic |  |  |  | 1.897(0.169) |
| Number of instruments |  |  |  | 2 |
| Endogeneity test |  |  |  | 3.281(0.070) |
| Cragg-Donald Wald F statistic |  |  |  | 285.860 |
| Kleibergen-paap rk LM statistic |  |  |  | 41.801(0.000) |
| Hausman |  | 80.37(0.000) |  |  |

**Notes:** Robust SEs and p-values are given in parentheses. *, ** and ***Denote significance at the 10%, 5% and 1% levels, respectively. MTCT rate of HIV and immunisation coverage are used as instruments for under-five mortality rate.
